# Relationships Between Maximal Aerobic Speed, Lactate Threshold, and Double Poling Velocity at Lactate Threshold in Cross-Country Skiers

**DOI:** 10.3389/fphys.2022.829758

**Published:** 2022-02-28

**Authors:** Jan-Michael Johansen, Arnstein Sunde, Jan Helgerud, Øyvind Støren

**Affiliations:** ^1^Department of Sports, Physical Education and Outdoor Studies, University of Southeastern Norway, Bø, Norway; ^2^Department of Circulation and Medical Imaging, Norwegian University of Science and Technology, Trondheim, Norway; ^3^Myworkout, Medical Rehabilitation Center, Trondheim, Norway

**Keywords:** maximal aerobic speed, aerobic endurance, endurance testing, cross-country skiing, lactate threshold

## Abstract

**Purpose:**

To investigate the relationships between maximal aerobic speed (MAS), lactate threshold in per cent of peak oxygen uptake (LT) and velocity at LT (LT_v_) in cross-country skiers. Secondly, we aimed to explore the fit of an equation previously used in cyclists and runners in a cohort of well-trained, competitive cross-country skiers for calculation of LT_v_. Thirdly, we aimed to investigate if a new LT_v_ could still be calculated after a period of regular training only by providing a new MAS.

**Methods:**

Ninety-five competitive cross-country skiers (65 males and 30 females) were tested for maximal oxygen uptake (VO_2max_), peak oxygen uptake in double poling (DP-VO_2peak_), oxygen cost of double poling (C_DP_), LT, and LT_v_. Thirty-five skiers volunteered to be tested 3 months later to evaluate potential changes in LT and LT_v_.

**Results:**

Velocity at LT was mainly determined by MAS (*r* = 0.88, *p* < 0.01). LT did not show a significant impact on LT_v_. The product of MAS·LT precisely predicted LT_v_ at baseline (*r* = 0.99, SEE = 2.4%), and by only measuring MAS, a new LT_v_ could be accurately calculated (*r* = 0.92, SEE = 6.8%) 3 months later in a sub-set of the initial 95 skiers (*n* = 35).

**Conclusion:**

The results suggest that LT has minor impact on LT_v_ in DP tested in a laboratory. LT_v_ seemed to be predominantly determined by MAS, and we suggest to put more focus on MAS and less on LT and LT_v_ in regular testing to evaluate aerobic performance capacity in DP.

## Introduction

Cross-country skiing is a demanding aerobic endurance sport (Sandbakk and Holmberg, 2017). The product of maximal oxygen uptake (VO_2max_) and oxygen cost of movement (C) has previously been established as maximal aerobic speed (MAS; McLaughlin et al., 2010; Støa et al., 2020), and a technique-specific MAS in double poling (DP) has shown strong associations to better DP performance in cross-country skiers (Sunde et al., 2019; Johansen et al., 2020, 2021).

Lactate threshold (LT) has been defined as the highest intensity where there is a balance between production and removal of blood lactate (Brooks, 1986), expressed as a percentage of VO_2max_ (Åstrand et al., 2003). Maximal lactate steady state (MLSS) is considered the most precise method to determine LT (Faude et al., 2009). As MLSS is a time-consuming method, several short-stage methods have been established and applied in previous studies, for example, a fixed LT at 4 mmol·L^−1^ (Green et al., 2006; Bragada et al., 2010; Rønnestad et al., 2014, 2016; Sylta et al., 2016) or an individual warm-up blood lactate concentration [La^−^]_b_ plus a constant (Helgerud et al., 1990; Støren et al., 2014; Skattebo et al., 2019; Støa et al., 2020). In previous studies in various aerobic endurance sports (McLaughlin et al., 2010; Støren et al., 2013; Laaksonen et al., 2020; Støa et al., 2020), no relationship was found between LT and aerobic endurance performance. In contrast, in previous studies of similar aerobic endurance sports, a significant relationship between endurance performance and the velocity at LT (LT_v_) has been observed (Bragada et al., 2010; McLaughlin et al., 2010; Støren et al., 2013; Johansen et al., 2020; Støa et al., 2020).

Although both VO_2max_ and C have been frequently studied, the relevance of LT in cross-country skiers has been less investigated. However, due to the constantly varying terrain, and thus high variation in intensity and speed, in traditional cross-country skiing events (Karlsson et al., 2018; Gløersen et al., 2020), a steady-state LT_v_ may not be of practical relevance. However, LT_v_ could be more relevant for steadier pace and intensity characteristics apparent in longer skiing events, for example, Vasaloppet (Stöggl et al., 2020).

Velocity at LT has previously been shown to be almost completely determined by the product of MAS and LT in running and cycling (Støren et al., 2014; Støa et al., 2020). In Støren et al. (2014), the following equation was used:


(1) $$LT_{w}=\left(LT,\;\%VO_{2\max}\right)\;VO_{2\max}/C_{c}$$

where LT_w_ is the power at LT, LT %VO_2max_ is the LT, and *C_c_* is the oxygen cost of cycling. This equation displayed a near-perfect correlation with LT_w_ measured with blood samples (*r* = 0.98, *SEE* = 2.8%). Only modified to running velocity, the same equation showed similar results in a large cohort of recreational to elite runners in Støa et al. (2020). In addition, in previous training interventions, improvements in LT_v_ or power at LT are often displayed together with improvements of either VO_2max_ or C, or both (e.g., Helgerud et al., 2001, 2007; Enoksen et al., 2011; Stöggl and Sperlich, 2014; Rønnestad et al., 2016).

Støren et al. (2014) suggest that by measuring MAS exclusively, a subsequent LT_v_ could be calculated, given no change in LT. Training interventions in already well-trained endurance athletes have displayed minor to no change in LT by training (Støren et al., 2008; Enoksen et al., 2011; Rønnestad et al., 2015, 2016; Sylta et al., 2016). Støa et al. (2020) displayed no differences in LT in runners ranging from a recreational to elite level. In contrast, improvements of VO_2max_ and C have frequently been observed (Sandbakk et al., 2013; Rønnestad et al., 2014; Johansen et al., 2021). The suggestion by Støren et al. (2014) has not yet been directly investigated in interventions.

Accordingly, we aimed to investigate the relationships of MAS and LT on LT_v_ in DP in cross-country skiers. Secondly, we aimed to explore the fit of the equation used in Støren et al. (2014) and Støa et al. (2020) on cyclists and runners, respectively, in a cohort of well-trained, competitive cross-country skiers for calculation of LT_v_. Thirdly, we aimed to investigate if a new LT_v_ could still be calculated after a period of regular training only by providing a new MAS.

## Materials and Methods

### Design

**Figure 1:**
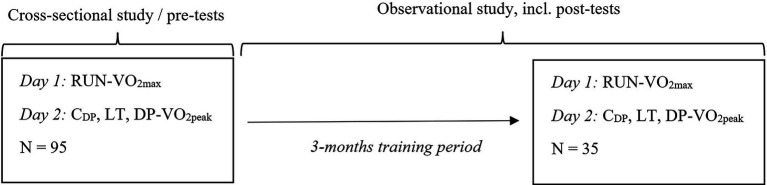
An overview of the study protocol.

The present results build upon parts of the data material in previously published works by our research group (Sunde et al., 2019; Johansen et al., 2020). However, the data analyzes, and study aims are not identical to these studies. The present study had both a cross-sectional design and an observational design with a pre- and a post-test, i.e., quasi-experimental. The participants that performed pre-tests were asked to voluntarily perform a post-test 3 months later. No interventional instructions were given during these 3 months, and the participants were asked to conduct their training as normal. The whole study protocol is depicted in Figure 1.

### Subjects

Ninety-five competitive and well-trained cross-country skiers (65 males and 30 females) were recruited and included for the baseline analyzes in the present study. Thirty-five of the participants volunteered to perform post-tests 3 months later. All tests were performed in the first half of the preparation period, i.e., April to September. The recruited skiers ranged in performance from mid-junior level to top national senior level. Subject and physiological characteristics are presented in Table 1.

**Table 1:** Subject characteristics at baseline.

Variable	All (*n* = 95)	Males (*n* = 65)	Females (*n* = 30)	*p*-value
Age (year)	20.0 ± 5.4 (27.0)	20.6 ± 6.1 (29.6)	18.7 ± 2.9 (15.5)	0.11
BW (kg)	71.3 ± 8.3 (11.6)	74.2 ± 7.7 (10.4)	65.1 ± 5.9 (9.1)	<0.01
Height (cm)	177.8 ± 8.2 (4.6)	181.9 ± 5.8 (3.2)	169.0 ± 5.2 (3.1)	<0.01
**RUN-VO_2max_**
mL·kg^−1^·min^−1^	65.5 ± 8.3 (12.7)	69.8 ± 5.7 (8.2)	56.3 ± 5.0 (8.9)	<0.01
L·min^−1^	4.70 ± 0.89 (19.0)	5.17 ± 0.63 (12.2)	3.69 ± 0.39 (10.6)	<0.01
**DP-VO_2peak_**
mL·kg^−1^·min^−1^	56.5 ± 7.8 (13.8)	60.4 ± 5.6 (9.3)	48.1 ± 4.3 (8.9)	<0.01
L·min^−1^	4.06 ± 0.84 (20.7)	4.48 ± 0.62 (13.8)	3.13 ± 0.39 (12.5)	<0.01
**C_DP_**
mL·kg^−1^·m^−1^	0.183 ± 0.021 (11.5)	0.177 ± 0.020 (11.3)	0.197 ± 0.020 (10.2)	<0.01
MAS (km·h^−1^)	18.8 ± 3.9 (20.7)	20.7 ± 3.2 (15.5)	14.7 ± 1.7 (11.6)	<0.01
**LT**
%DP-VO_2peak_	81.4 ± 6.7 (8.2)	80.7 ± 7.0 (8.7)	82.9 ± 5.9 (7.1)	0.15
*v* (km·h^−1^)	15.3 ± 2.7 (17.6)	16.6 ± 1.9 (11.4)	12.3 ± 1.5 (12.2)	<0.01
**Calc. LT_v_**
MAS·LT_%_	15.2 ± 2.8 (18.4)	16.6 ± 2.0 (12.0)	12.2 ± 1.5 (12.3)	<0.01

*Values are mean and SD with coefficient of variance in percentage in parenthesis. *p* value indicates the level of significance for the difference between males and females. yr, years; BW, body weight; kg, kilograms; RUN-VO_2max_, maximal oxygen uptake in running; mL·kg^−1^·min^−1^, milliliters oxygen per kilogram bodyweight per minute; L min^−1^, liters per minute; DP-VO_2peak_, peak oxygen uptake in double poling; C_DP_, oxygen cost of double poling at 80% of DP-VO_2peak_; mL·kg^−1^·m^−1^, milliliters oxygen per kilogram body weight per meter; MAS, maximal aerobic speed; km·h^−1^, kilometers per hour; LT, lactate threshold; %DP-VO_2peak_, LT percentage of DP-VO_2peak_; v, velocity; and Calc. LT_v_, calculated lactate threshold velocity by Equation 2.*

The study was conducted in accordance with the Declaration of Helsinki, and the regional ethical committee (REC) and the ethical board at the University of Southeastern Norway approved the study. All participants gave their written informed consent after being provided oral and written information about the study protocol.

### Methodology

All physiological tests were conducted over 2 consecutive days at the same laboratory. The first test day included a VO_2max_test in running (RUN-VO_2max_). The second test day consisted of a sub-maximal DP-test on a treadmill to measure LT, LT_v_, and oxygen cost of DP (C_DP_), and an incremental peak oxygen uptake in DP (DP-VO_2peak_) test. All preparation procedures prior to testing are described previously (Sunde et al., 2019; Johansen et al., 2020). In short, the protocol for this was that only easy training, i.e., training below 70% of HR_max_, was allowed the last 24 h before tests. Food intake and nutritious drinks were not allowed the last hour prior to testing both days, while water intake was allowed. For the participants attending to the laboratory 3 months later, identical procedures were followed.

#### Day 1

The first day of testing started with a self-conducted warm-up procedure of 10 min, before the incremental RUN-VO_2max_ test. The test was performed on a Woodway PPS 55 sport (Waukesha, WI, United States), controlled for speed and incline. The starting intensity was set to 7–8 and 9–10 km·h^−1^ for females and males, respectively, and 6% inclination. Within the first minute of the test, inclination increased to 8%, while only speed was increased by 0.5 km·h^−1^ every 30 s after that. The test terminated at voluntary fatigue, and VO_2max_ was calculated by the three highest consecutive VO_2_measurements. In addition to voluntary fatigue and flattening of the VO_2_ curve, heart rate at ≥98% of HR_max_, RER ≥ 1.05, and rate of perceived exertion ≥17 (Borg scale 6–20) was used to evaluate if VO_2max_ was achieved. All VO_2_ measurements were performed with a MetaLyzer Cortex II (Biophysic GmbH, Leipzig, Germany), with measurements every 10 s. Prior to all tests, flow sensors were calibrated with a 3-L calibration syringe, and O_2_ analyzers were calibrated with certified calibration gases (16% O_2_/4% CO_2_) and ambient air. All HR measurements were performed with Polar s610 monitors (Kempele, Finland) or the participants individual HR monitor.

#### Day 2

Prior to the LT, LT_v_, and C_DP_ assessments, all participants were familiarized to the rollerskiing treadmill (Rodby RL 2700E, Rodby Innovation, Vänge, Sweden) with a 30-min workout on different skiing velocities. After termination of the treadmill familiarization, measurements of [La^−^]_b_, heart rate (HR), and oxygen consumption (VO_2_) were measured in 4-min work periods while double poling at different sub-maximal intensities to evaluate LT, LT_v_, and C_DP_. The first work period started with an intensity assumed to be approximately 60% of DP-VO_2peak_. This corresponded to 4% inclination and 10–11.5 km·h^−1^ for males and 6–8 km·h^−1^ for females. After the first work period, the velocity was increased by 1–3 km·h^−1^ for subsequent work periods until [La^−^]_b_ values exceeded the LT values. The individual LT value was calculated by the individual warm-up lactate value +2.3 mmol·L^−1^. This protocol is presented and evaluated in Helgerud et al. (1990) and Medbø et al. (2000) and previously used in several studies (Helgerud et al., 2007; Støren et al., 2013, 2014; Sunde et al., 2019; Johansen et al., 2020; Støa et al., 2020). The mean VO_2_ during the last minute of every work period was used to calculate C_DP_ and LT. A Lactate Scout+ (SensLab, GmbH, Leipzig, ray Inc., Kyoto, Japan) was used to measure whole blood lactate values.

A DP-VO_2peak_test was performed 5 min after the LT and C_DP_ assessments. For 47 participants, the protocol presented in Sunde et al. (2019) was used. Briefly, the starting intensity was set to 6% inclination and 6 and 11.5 km·h^−1^ for females and males, respectively. Every 30 s, the speed was increased by 1 km·h^−1^ until 10 and 18 km·h^−1^ was reached for females and males, respectively. Increments of 0.5 km·h^−1^ every 30 s were then provided until exhaustion. For the remaining participants (*n* = 48), the protocol presented in Johansen et al. (2020) was used. The starting intensity was set to 7 km·h^−1^ and 6% inclination. While inclination was held constant, the speed increased by 1 km·h^−1^ every minute until exhaustion for both genders. In both protocols, voluntary exhaustion was defined as the time where the participants no longer were able to maintain their position at the treadmill. The test terminated when the subjects reached a pre-defined mark 1 m behind the starting position on the treadmill. By both protocols, the DP-VO_2peak_ was defined as the mean of the two highest consecutive VO_2_measurements. MAS was calculated as the product of DP-VO_2peak_ divided by C_DP_ at 80% of DP-VO_2peak_ for all participants. To calculate LT_v_, an equation based on Støren et al. (2014) and Støa et al. (2020) was used:


(2) $$LT_{v}=LT\left(DP-VO_{2\mathrm{peak}}/C_{DP}\right)$$

where LT_v_ is the velocity at LT, LT is LT in percent of DP-VO_2peak_, and C_DP_ is the oxygen cost of DP.

### Statistical Analysis

The data material was found to be normally distributed by use of quantile– quantile (QQ) plots and normality tests for DP-VO_2peak_, C_DP_, and LT. Thus, parametric statistics were used. Mean ± SD and inter-individual variability expressed as coefficient of variance (CV) were used as descriptive statistics for all variables. Independent sample t-tests and paired sample t-tests were used to evaluate differences between genders at baseline and physiological differences from pre to post (*n* = 35), respectively. To evaluate possible relationships between variables at baseline and changes in variables over time (delta correlations), correlation coefficients *r* was used from Pearson’s bivariate tests. Since the data material consisted of both males and females, partial correlations corrected for gender were also performed. Standard error of the estimate (SEE) was calculated by linear regression analyses.

In all two-tailed tests, the level of significance was set to a value of *p* < 0.05. The statistical package for social science, version 26 (SPSS, IBM, Chicago, IL, United States) was used for all statistical analyses.

## Results

Males had significantly higher values in all physiological variables, with two exceptions where males had significantly better C_DP_ compared to females while no difference was observed in LT (Table 1). Equation 2 for calculating LT_v_ displayed an almost identical value as the lactate measured LT_v_ for the whole group, in males and in females. For the 35 skiers performing post-tests, no variables changed significantly (Table 2).

**Table 2:** Physiological characteristics prior and post 3 months of regular training (*n* = 35).

Variable	Pre		Post	
BW (kg)	70.2 ± 8.9	(13.5)	70.0 ± 8.9	(12.8)
**DP-VO_2peak_**
mL·kg^−1^·min^−1^	55.1 ± 7.6	(14.2)	55.8 ± 7.9	(14.2)
L min^−1^	3.89 ± 0.86	(22.1)	3.92 ± 0.86	(21.9)
HR	191.0 ± 9.5	(5.0)	190.8 ± 9.6	(5.0)
RER	1.10 ± 0.05	(4.9)	1.10 ± 0.05	(4.6)
RPE	17.6 ± 1.2	(7.1)	17.6 ± 1.1	(6.4)
**C_DP_**
mL·kg^−1^·m^−1^	0.196 ± 0.021	(10.5)	0.193 ± 0.018	(9.5)
**MAS (km·h^−1^)**
MAS	17.2 ± 3.4	(19.38)	17.5 ± 3.2	(18.4)
**LT**
%DP-VO_2peak_	82.6 ± 6.1	(7.4)	82.8 ± 6.3	(7.6)
v (km·h^−1^)	14.2 ± 2.7	(19.1)	14.6 ± 2.4	(16.7)
**Calc. LT_v_**
MAS·LT_%_	14.1 ± 2.7	(19.3)	14.4 ± 2.7	(18.6)

Values are mean and SD with coefficient of variance in percentage in parenthesis. BW, body weight; kg, kilograms; DP-VO_2peak_, peak oxygen uptake in double poling. mL·kg^−1^·min^−1^, milliliters oxygen per kilogram bodyweight per minute; L min^−1^, liters per minute; HR, heart rate; RER, respiratory exchange ratio; RPE, rate of perceived exertion; C_DP_, oxygen cost of double poling at 80% of DP-VO_2peak_; mL·kg^−1^·m^−1^, milliliters oxygen per kilogram per meter; MAS, maximal aerobic speed calculated as the product of DP-VO_2peak_ and C_DP_ at 80%; LT, lactate threshold; %DP-VO_2peak_, LT percentage of peak oxygen uptake in DP; v, velocity; km, kilometers; h, hours; and Calc. LTv, calculated lactate threshold velocity.

**Figure 2:**
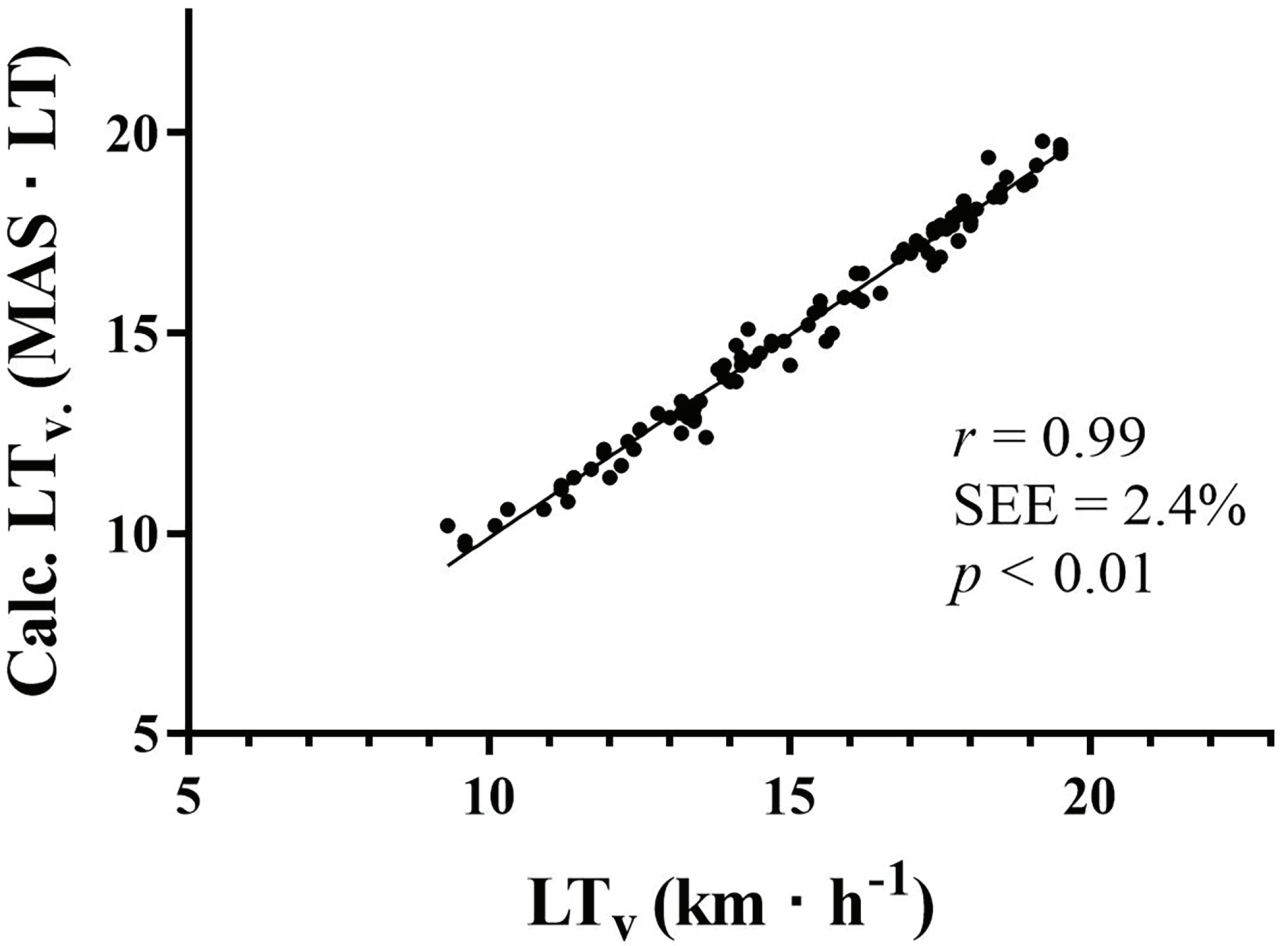
Baseline correlation between velocity at LT (LT_v_) measured by blood lactate and LT_v_ predicted by Equation 2. LT_v_, velocity at lactate threshold; Calc. LT_v_, calculated LT_v_ by Equation 2; km·h^−1^, kilometers per hour; *r*, correlation coefficient; and SEE, standard error of the estimate in percentage.

**Figure 3:**
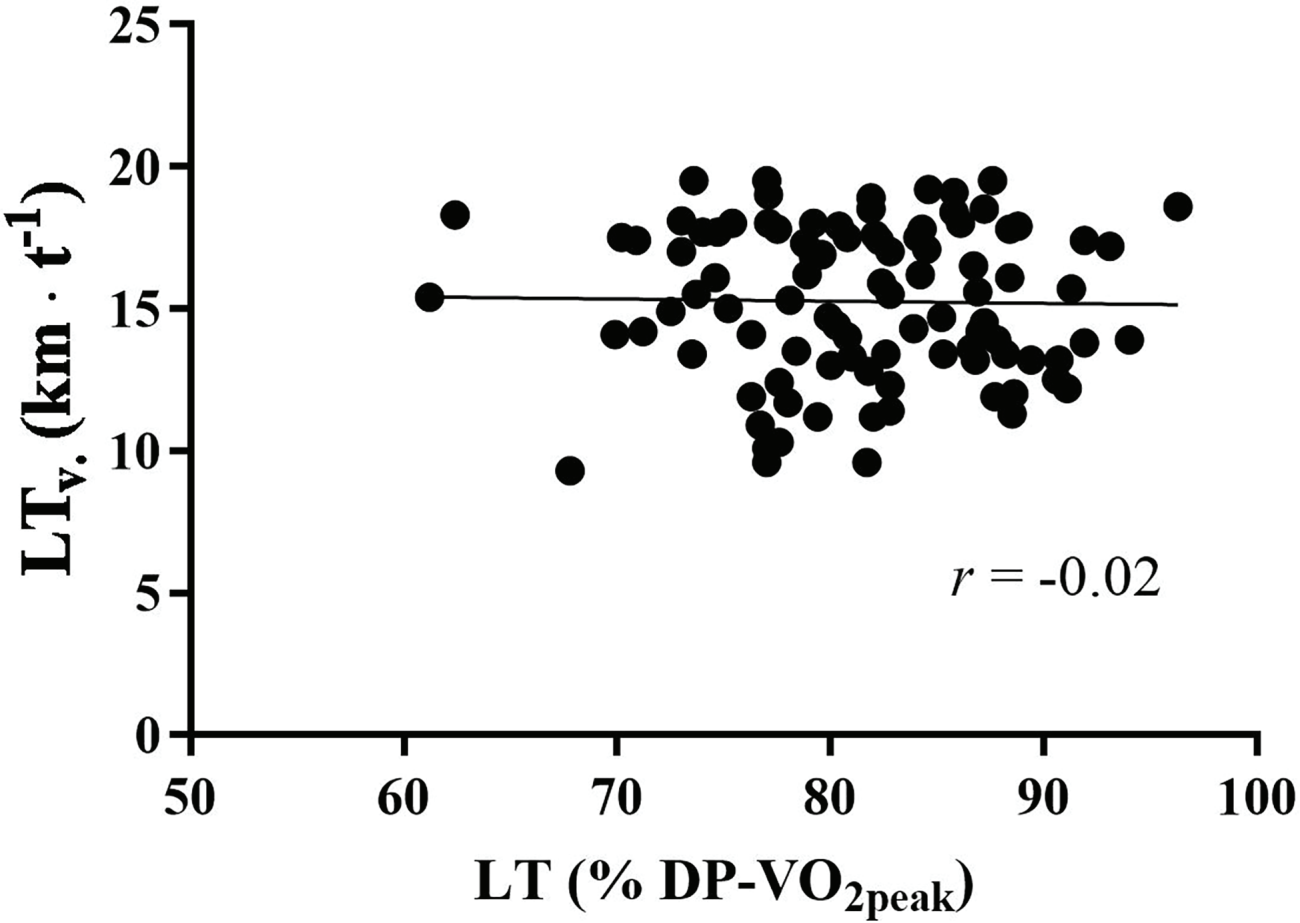
Baseline correlation between LT_v_ and LT. LT_v_, velocity at lactate threshold. LT, lactate threshold as a percentage of DP-VO_2peak_; km·h^−1^, kilometers per hour; and *r*, correlation coefficient.

Baseline correlations are presented in Table 3. The product of MAS·LT correlated very strongly with lactate measured LT_v_ (Figure 2) and was held equally strong when controlled for gender and analyzed within genders. By use of this equation, LT_v_ could be predicted within a range of 0.4 km·h^−1^. MAS displayed strong correlations to LT_v_ (*r*^2^ = 0.77, SEE = 8.5%) independent of genders, while LT did not show any correlation at all (Figure 3). Both DP-VO_2peak_ (*r*^2^ = 0.71, SEE = 9.8%) and C_DP_ (*r*^2^ = 0.36, SEE = 14.4%) correlated significantly with LT_v_.

**Table 3:** Baseline correlations with LT_v_.

Variable	All pooled (*n* = 95)	Controlled for gender (*n* = 95)	Within genders	
			Males (*n* = 65)	Females (*n* = 30)
**DP-VO_2peak_**
mL·kg^−1^·min^−1^	0.84**	0.66**	0.67**	0.55**
L·min^−1^	0.81**	0.69**	0.59**	0.40*
HR	−0.08	−0.24	−0.08	−0.01
RER	−0.06	−0.09	−0.25*	−0.31
RPE	0.04	0.25	0.28*	−0.39*
**C_DP_**
mL·kg^−1^·m^−1^	−0.60**	−0.47**	−0.52**	−0.36
**MAS (km·h^−1^)**
MAS	0.88**	0.88**	0.74**	0.75**
**LT**
%DP-VO_2peak_	−0.02	<0.01	0.04	0.47**
*v* (km·h^−1^)	-	-	-	-
**Calc. LT_v_**.
MAS·LT%	0.99**	0.97**	0.98**	0.98**

*Values are correlation coefficient r for all participants pooled, partial correlations controlled for gender and within genders. DP-VO_2peak_, peak oxygen uptake in double poling; mL·kg^−1^·min^−1^, milliliters per kilogram bodyweight per minute; L·min^−1^, liters per minute; HR, heart rate; RER, respiratory exchange ratio; RPE, rate of perceived exertion; C_DP_, oxygen cost of double poling at 80% of DP-VO_2peak_; mL·kg^−1^·m^−1^, milliliters per kilogram per meter; MAS, maximal aerobic speed calculated as the product of DP-VO_2peak_ and C_DP_ at 80%; LT, lactate threshold; %DP-VO_2peak_, LT percentage of peak oxygen uptake in DP; *v*, velocity; km, kilometers; h, hours; and Calc. LT_v_; calculated lactate threshold velocity*.

* *p < 0.05 significant correlation*.

** *p < 0.01 significant correlation*.

**Figure 4:**
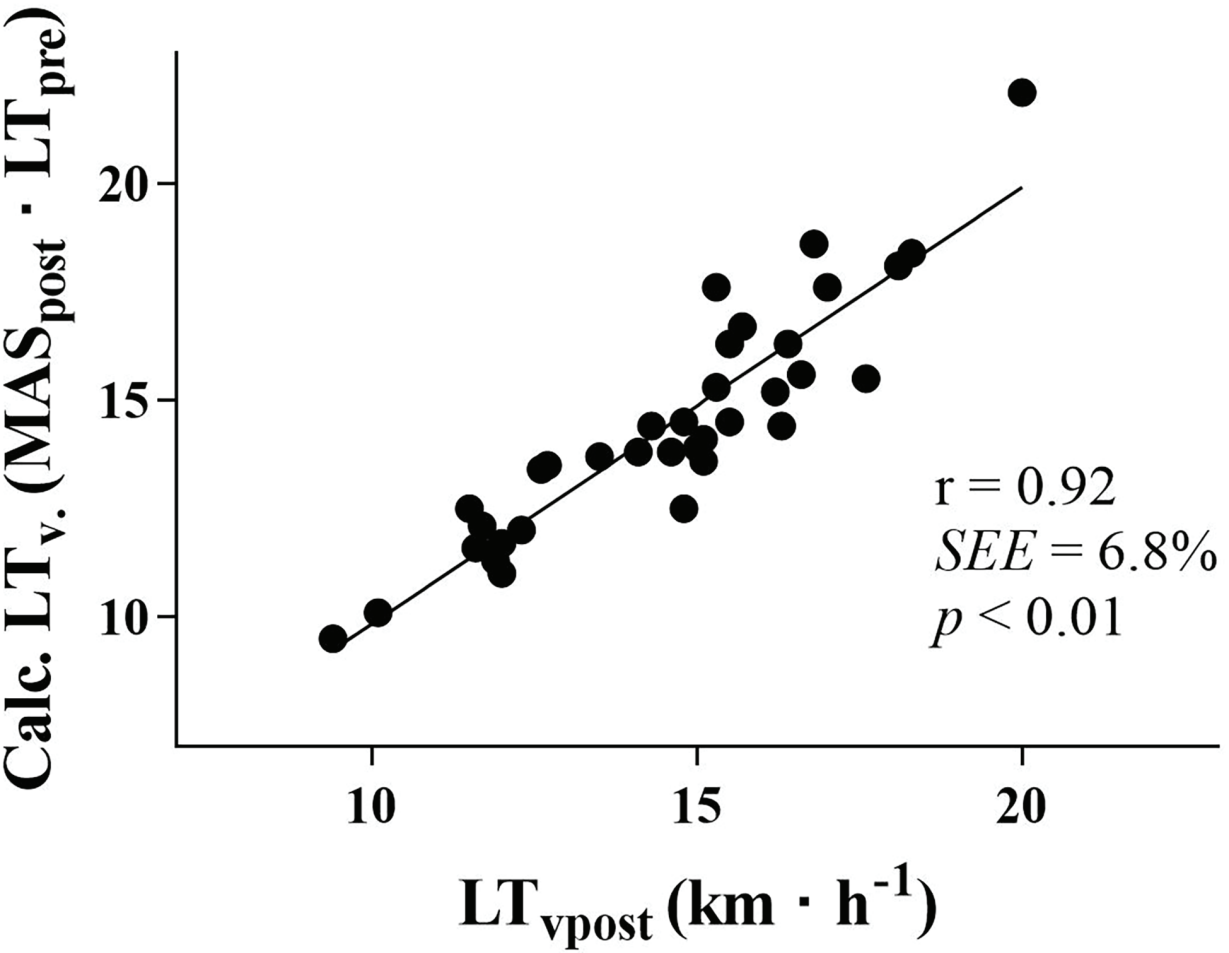
Correlation between LT_v_ at post-test measured by blood lactate and calculated LT_v_. LT_vpost_, velocity at lactate threshold at post-test. LT_v_ pred., calculated LT_v_ by a new MAS and LT from pre-tests. km·h^−1^, kilometers per hour; *r*, correlation coefficient; and SEE, standard error of the estimate in percentage.

**Figure 5:**
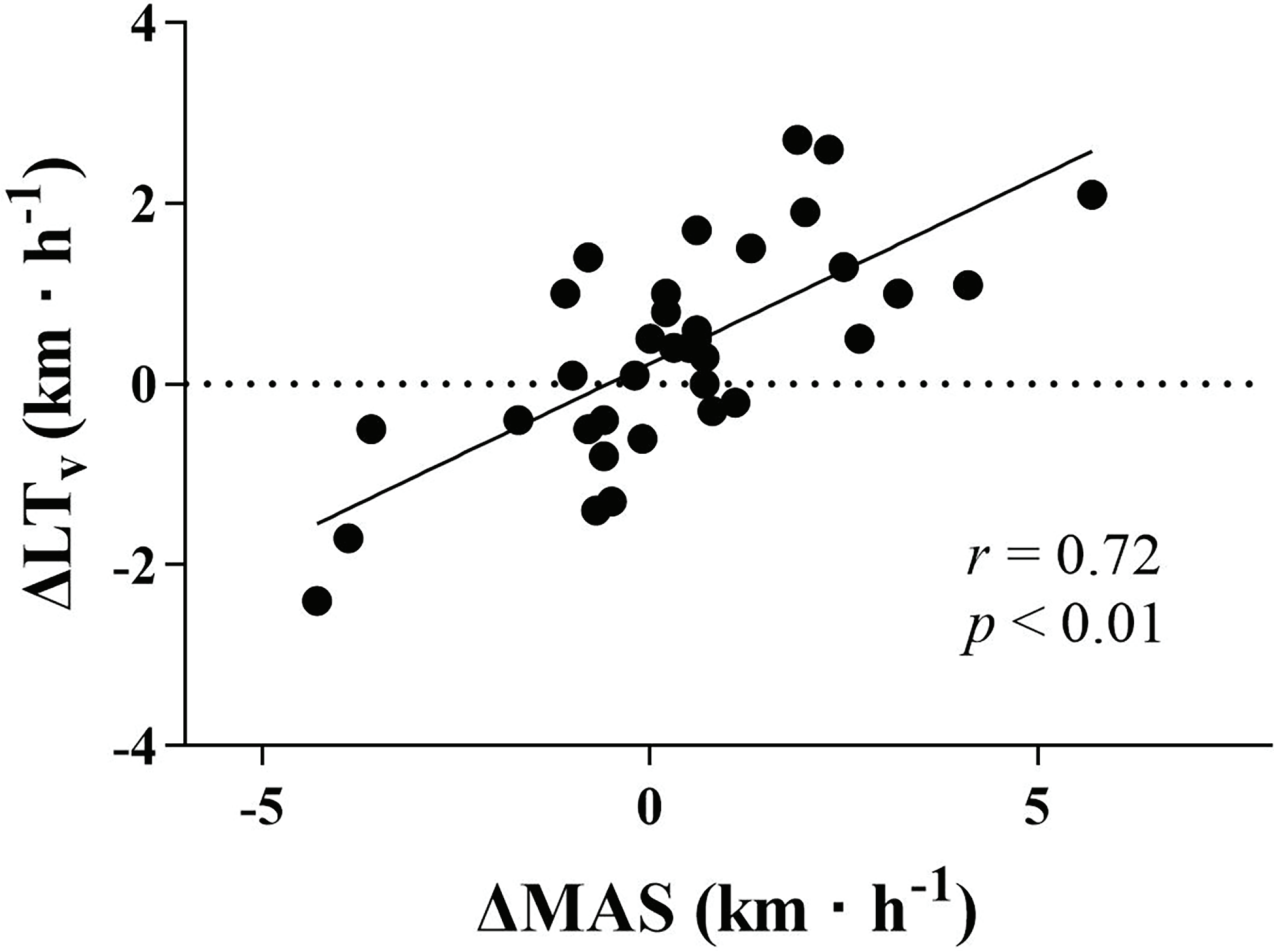
Delta correlation between change in MAS and change in LT_v_. ∆LT_v_; change in velocity at lactate threshold; ∆MAS, change in maximal aerobic speed; km·h^−1^, kilometers per hour; and *r*, correlation coefficient.

By the use of the previously measured LT and a new MAS (MAS_post_·LT_pre_). LT_v_ could be calculated at post-test within a range of 1.0 km·h^−1^ (Figure 4). Delta correlations also revealed a strong relationship between ΔLT_v_ and ΔMAS (Figure 5). In addition, the changes in calculated LT_v_ followed the changes in lactate measured LT_v_ (*r* = 0.74, *p* < 0.01). In line with the baseline correlations, no significant delta correlation was apparent between ΔLT_v_ and ΔLT (*r* = 0.11). However, ΔMAS correlated moderately negatively with ΔLT (*r* = −0.50, *p* < 0.01), which means that an improved MAS resulted in a lower LT and a reduced MAS provided higher LTvalues.

During the 3-month training period, the 35 skiers that volunteered for post-tests trained a total of 12.1 ± 3.0 h·week^−1^. Of this, 10.5 ± 2.8 h·week^−1^ were performed as endurance training. The relative intensity distribution was 77.8 ± 7.2% below 82% of HR_max_, 4.2 ± 1.8% at 82%–87% HR_max_, and 4.6 ± 2.4% above 87% of HR_max_. Additionally, 10.1 ± 4.3% was performed as strength, speed and jump training, while 3.3 ± 4.9% was other training, e.g., soccer.

## Discussion

The main findings of the present study were (1) that the same equation as in Støren et al. (2014) and Støa et al. (2020) calculated LT_v_ precisely in a large group of competitive cross-country skiers, (2) that MAS showed a major impact on LT_v_ both at baseline and over time, (3) that LT was not associated with LT_v_, and (4) that only by measuring MAS, a subsequent LT_v_ could be strongly predicted after 3 months of regular training.

In the present study, LT_v_ was precisely calculated within a range of 0.4 km·h^−1^ (Figure 2) by the proposed mathematical model (Equation 2), and this finding supports previous use of this equation (Støren et al., 2014; Støa et al., 2020). Furthermore, variation in LT_v_ seemed to follow variations in MAS, given the strong correlation between these two variables (*r* = 0.88). In contrast, LT did not show a significant impact on LT_v_. These results support previous findings in large cohorts of competitive cyclists (Støren et al., 2014) and runners (Støa et al., 2020) that LT_v_ is determined by MAS. This assumption is further strengthened by the significant correlation between ∆MAS and ∆LT_v_ in the present study. Previous training interventions also support the strong determinant impact of MAS on LT_v_, since improvements in LT_v_ are frequently observed together with increased MAS (Helgerud et al., 2001, 2007; Enoksen et al., 2011).

Støren et al. (2014) suggested that a subsequent LT_v_ could be calculated only by measuring MAS at subsequent testing sessions, given no change in LT. Several studies have reported minor to no adaptations in LT by training in already well-trained endurance athletes (Støren et al., 2008; Enoksen et al., 2011; Rønnestad et al., 2015, 2016; Sylta et al., 2016), while we have not been able to find reports with a significantly improved LT. To our knowledge, the present study is the first study to apply the calculated LT_v_ in DP both pre and post a regular training period. However, the regular training performed by the skiers in this period resulted in only small non-significant changes in DP-VO_2peak_, C_DP_, LT, and LT_v_. No significant changes were detected in RUN-VO_2max_, RER or BW either (Table 2). Still, by measuring MAS at post-test in these skiers, and use the initial LT, LT_v_ was predicted accurately within a range of 1.0 km·h^−1^ (Figure 4) compared to blood lactate measured LT_v_. This supports the suggestion made by Støren et al. (2014), although some caution should be taken in interpreting results with so small training adaptations.

Since LT_v_ has been shown to depend primarily on MAS in the present study, as similar to the findings in Støren et al. (2014) and Støa et al. (2020), the impact of LT_v_ on aerobic endurance performance (Støren et al., 2008, 2013; Bragada et al., 2010; McLaughlin et al., 2010; Johansen et al., 2020; Støa et al., 2020) may be regarded just as much as a result of MAS, as an independent performance-determining variable. LT, thus as a percent of VO_2max_ or VO_2peak_, has on the other hand not been found to change much with training, nor has it been found to be a strong and independent performance-determining variable (Støren et al., 2008, 2013; McLaughlin et al., 2010; Johansen et al., 2020; Støa et al., 2020). In Støa et al. (2020), no difference was observed in LT among runners at a recreational to elite level. We therefore call for a discussion on whether or not regular LTassessments should be a useful tool to evaluate aerobic endurance performance level and training adaptations and if LT should be considered one of the main performance-determining variables in aerobic endurance sports (Pate and Kriska, 1984; Joyner and Coyle, 2008).

By only testing for VO_2max_ or technique-specific VO_2peak_ and C, the present findings combined with similar research (Støren et al., 2014; Støa et al., 2020) suggest that endurance athletes could generate a proper estimation of their development of LT_v_ and aerobic endurance performance capacity, at least over shorter time frames. A suggestion for a possible testing protocol for cross-country skiers could be to perform an initial assessment of LT with continuous VO_2_ measurements to determine the per cent of VO_2max_/VO_2peak_, LT_v_, and C in the utilized technique, i.e., DP, and followed by a VO_2max_/VO_2peak_ test like that of the present study. The whole protocol lasts approximately 45 min per athlete. For a subsequent test, only a 5- to 10-min warm-up procedure, 1–2 sub-maximal work periods to determine C at the preferable intensity (e.g., 80% VO_2max_), and a VO_2max_/VO_2peak_ test are needed to calculate LT_v_. This protocol may not last for more than 20–25 min. Consequently, competitive cross-country skiers could reduce time for physiological testing of aerobic variables by approximately 50%.

The impact of LT_v_ on aerobic endurance performance has been reported to be high in several studies in running (Bragada et al., 2010; McLaughlin et al., 2010; Støa et al., 2020) and cycling (Støren et al., 2013; Heuberger et al., 2018). However, the practical relevance of LT_v_ in a single sub-technique in cross-country skiing could be questioned. The variation in terrain imposes variations in sub-techniques dependent on both speed and intensity over a traditional cross-country skiing competition (i.e., World Cup or World Championships; Karlsson et al., 2018; Gløersen et al., 2020). However, longer skiing events, like e.g., Vasaloppet and Marcialonga, are characterized with less variation in sub-techniques as well as in terrain (Stöggl et al., 2020). The performance in such competitions may in turn rely more on a steady sub-maximal velocity in a single sub-technique. These long-distance skiing competitions are thus more like long-distance running, characterized by a steadier sub-maximal race velocity and less variations in techniques (Ely et al., 2008).

### Practical Implications and Limitations

Velocity at LT was found to be primarily a product of MAS in the present study. To evaluate training adaptations, instead of testing for LT, we suggest regular tests of VO_2max_ or technique-specific VO_2peak_ and C. The latter would also save time and blood samples.

The large number of participants in the present study makes the results statistically strong. However, controlled training interventions aimed to impose significant adaptations in either VO_2peak_ or C or both should be performed to potentially confirm causal relationships between MAS, LT, and aerobic endurance performance. The proposed mathematical model utilized for calculations of LT_v_ and suggested testing protocol for LT over time in the present study is only valid as long as LT does not change. Although we were not able to find scientific evidence of large adaptations in LT and that the present study did not reveal any adaptations in LT, we cannot completely rule out that this might be possible over longer time frames with substantial changes in performance level and in other less trained athletes. Thus, investigations of longer time span and in different cohorts of cross-country skiers (elite and/or recreational athletes) should be performed to confirm the present findings. In addition, evaluation of these relationships and the direct impact on performance in longer cross-country skiing events (10–90 km) and in other classical and skating techniques are warranted.

### Conclusion

Both baseline and adaptations in LT_v_ were minorly influenced by LT and merely a product of MAS. In addition, only by providing new MAS, a subsequent LT_v_ could be strongly predicted after 3 months of training.

## Data Availability Statement

The raw data supporting the conclusions of this article will be made available by the authors, without undue reservation.

## Ethics Statement

The studies involving human participants were reviewed and approved by Regional Ethics Committee of Southeastern Norway and the Institutional Research Board at the University of Southeastern Norway. Written informed consent to participate in this study was provided by the participants. For all participants under the age of 18, written informed consent was provided from their legal guardian/next of kin.

## Author Contributions

All authors contributed significantly to the design and planning of the study. ØS and J-MJ led the interpretation of data, while J-MJ led the writing of the manuscript. J-MJ, ØS, and AS took part in the data collection. All authors contributed to the different data analyzes and edited, reviewed, and approved the manuscript.

## Conflict of Interest

The authors declare that the research was conducted in the absence of any commercial or financial relationships that could be construed as a potential conflict of interest.

## Publisher’s Note

All claims expressed in this article are solely those of the authors and do not necessarily represent those of their affiliated organizations, or those of the publisher, the editors and the reviewers. Any product that may be evaluated in this article, or claim that may be made by its manufacturer, is not guaranteed or endorsed by the publisher.
